# Trying hard not to listen: the evolution of information processing in vestibular hair cells

**DOI:** 10.1186/1471-2202-14-S1-P313

**Published:** 2013-07-08

**Authors:** Alessandro Venturino, Martina Rizza, Matteo Pedrazzoli, Paola Perin

**Affiliations:** 1 Department of Brain and Behavioural Sciences, University of Pavia, Pavia, 27100 Italy

## 

Vestibular afferents encode head movements with a discharge that follows stimulus timecourse. In aquatic animals, both vestibular and auditory stimuli are carried by water, and mainly differ in spectral power content. For land animals, sounds are carried by air vibration that minimally affect head movements, so that auditory and vestibular organs receive separate inputs. It is therefore no surprise that auditory and vestibular hair cells are much more similar in fish than in tetrapods. In this work we compare the two different strategies for encoding vestibular signals by hair cells in lower and higher vertebrates, and suggest a possible pathway for the evolution of the vestibular organs (by modeling the electrical membrane changes necessary and sufficient to transform one encoding strategy into the other).

Amphibia represent a primitive transition to land life, and their inner ear is primitive in many ways, containing organs, such as the saccule, which cannot be classified as purely auditory nor vestibular. However, it also displays proper vestibular organs, namely an utricle, a lagena and three semicircular canals. For each vestibular organ, afferents display variations in gain, dynamics, resting discharge frequency and regularity which are similar to those in higher vertebrates, in particular a low-gain, phasic population is observed, similar to fibers innervating type-I hair cells in amniotes. On the other hand, hair cells still show primitive features, namely the presence, in the population connecting to phasic fibers, of resonant behaviour due to a Ca-BK system, which is absent in higher vertebrates, that instead possess type I hair cells, which display a characteristic large M current which profoundly change their electrophysiological properties.

We have investigated the membrane and release properties of frog utricular and semicircular canal hair cells, by recording (with perforated patch-clamp) current, voltage and release from hair cells differing in morphology and epithelial position. Data from these experiments, together with literature data for buffer expression and afferent innervation, and immunohistochemistry data for intracellular Ca sources, were used to build a NEURON model of utricular hair cells reproducing literature data for afferent discharge. Our model show that Ca-BK resonance of phasic hair cells, which is set at higher frequencies than vestibular stimuli, imparts a phase advance to hair cell release relative to the mechanical stimulus, but also introduces other linearity distortions, such as gain compression, which are not present in fibers connecting to type I hair cells. Assuming that during evolution from amphibia to reptiles no intermediate animal could survive without a working vestibular system, we explored (with NEURON models) possible transitions from a resonant, ICa-based cell to a nonresonant, IM-based cell that allowed phasic transduction in all intermediate stages.

